# MicroRNA miR-193b-3p Regulates Esophageal Cancer Progression Through Targeting RSF1

**DOI:** 10.3390/cells14120928

**Published:** 2025-06-19

**Authors:** Yao Lin, Xudong Zhao, Zhenhua Du, Zhili Jia, Siyu Zhou, Gengsheng Cao, Hengbin Wang

**Affiliations:** 1 School of Life Sciences, Henan University, Kaifeng 475004, China; linyusen@henu.edu.cn (Y.L.); zhaoxudong@connect.hku.hk (X.Z.); hzdw0324@163.com (Z.D.); chilejia@163.com (Z.J.); zhou4yu@163.com (S.Z.); 2 Department of Obstetrics and Gynecology, Li Ka Shing Faculty of Medicine, The University of Hong Kong, Hong Kong 999077, China; 3 Department of Biochemistry and Molecular Genetics, University of Alabama at Birmingham, Birmingham, AL 35294, USA; 4 Department of Internal Medicine, Division of Hematology, Oncology and Palliative Care, Massey Comprehensive Cancer Center, Virginia Commonwealth University, Richmond, VA 23298, USA

**Keywords:** esophageal cancer, microRNA, miR-193b-3p, RSF1, cancer progression

## Abstract

Esophageal cancer (ESCA) is the sixth leading cause of cancer-related mortality worldwide. Despite the significant impact, the molecular mechanisms underlying its initiation and progression remain poorly understood. In this study, we identified mircoRNA miR-193b-3p as a critical regulator of ESCA progression and the Remodeling and Spacing Factor 1 (RSF1) as an essential target of miR-193b-3p. Analysis of the TCGA_ESCA dataset and RT-qPCR experiments revealed that RSF1 levels are significantly elevated in ESCA and inversely correlated with miR-193b-3p levels. Using a dual-luciferase reporter assay, as well as transfection of miR-193-3p mimics or inhibitors, we confirmed RSF1 as a direct target of miR-193b-3p in ESCA cells. Transfection of miR-193b-3p suppresses ESCA cell proliferation, migration, and invasion. These effects were partially reversed by exogenous RSF1 expression. Injection of AgomiR-193b-3p into mice bearing ESCA xenografts impeded tumor growth. These findings underscore the critical role of the miR-193b-3p/RSF1 axis in esophageal cancer progression.

## 1. Introduction

Esophageal cancer (ESCA) is a common malignancy of the upper digestive system, clinically classified into two main subtypes: esophageal adenocarcinoma (EAC) and esophageal squamous cell carcinoma (ESCC). ESCC accounts for over 90% of ESCA cases in high-risk regions, such as China, whereas EAC predominates in Western countries [1]. The development of ESCA is influenced by a combination of genetic predispositions, environmental factors, and lifestyle choices. Frequently mutated genes, such as TP53 [2], NOTCH1 [3], and PIK3CA [4], contribute to disease predisposition, while epigenetic alternations, including DNA methylation and histone modifications, lead to the silencing of tumor suppressor genes and contribute to disease progression [5,6]. Environmental and lifestyle factors, such as smoking, alcohol consumption, intake of hot foods, nutritional deficiencies (e.g., zinc and selenium), and chronic esophagitis, further accelerate ESCA development. In China, the burden of ESCC is particularly high, accounting for approximately 50% of global cases [7]. Regions such as Henan, Hebei, and Shanxi are among the most affected, largely due to specific local environmental conditions and lifestyle practices [8]. ESCA is often diagnosed at advanced stages, characterized by aggressive tumor growth, limited treatment options, and poor overall patient health [9]. Current therapeutic approaches, including surgery, radiation, chemotherapy, and targeted therapies, often yield limited efficacy and are accompanied by significant side effects. As a result, the 5-year survival rate remains as low as approximately 20% [10]. These challenges emphasize the urgent need for a better understanding of the disease progression and more effective and personalized treatments.

RSF1 (Remodeling and Spacing Factor 1) is a critical component of the RSF chromatin remodeling complex, which collaborates with SNF2H (SMARCA5), an ATPase of the ISWI (Imitation Switch) family of chromatin remodelers [11]. RSF1 plays a critical role in maintaining chromatin structures, regulating transcription, and facilitating DNA replication and repair by controlling nucleosome assembly and spacing [12,13,14]. Our previous studies have demonstrated that RSF1 specifically interacts with ubiquitinated histone H2A nucleosomes (H2Aub) through a unique ubiquitinated H2A binding (UAB) domain (amino acid 770–807). In so doing, RSF1 regulates genes that overlap with those controlled by the Polycomb Repressive Complex 1 (PRC1). Knockout of RSF1 results in the disruption of stable nucleosome arrays at promoter regions, underscoring its vital role in H2Aub-mediated gene silencing [15]. Overexpression of RSF1 is frequently observed in various cancers and is often associated with poor prognosis, enhanced tumor aggressiveness, and therapy resistance [12]. For instance, in ovarian cancer, elevated RSF1 levels have been implicated in resistance to paclitaxel, a commonly used chemotherapeutic drug, thereby reducing its efficacy [16]. Similarly, in hepatocellular carcinoma, elevated RSF1 levels have been linked to malignant progression through its influence on key cell cycle checkpoints, leading to uncontrolled cell division and tumor growth [17]. Moreover, inhibition of RSF1 has been shown to reduce the proliferation of cancer cells [18], further supporting the notion that elevated RSF1 levels promote cell survival and tumor growth [19]. Despite these advances, the role of RSF1 in the pathogenesis and progression of ESCA remains largely unexplored.

MicroRNAs (miRNAs) are endogenously synthesized non-coding RNAs, typically 18–24 nucleotides in length, that regulate gene expression at post-transcriptional levels. miRNAs bind to the 3′ untranslated region (3′ UTR) of target mRNAs, leading to mRNA degradation or translational repression [20]. Since their discovery, miRNAs have been recognized as critical regulators of various cellular processes, including development, differentiation, apoptosis, and proliferation [21]. MicroRNA (miRNA) expression profiles are characterized by tissue specificity arising from factors such as their genomic localization within host genes, transcriptional regulation by tissue-specific promoters, epigenetic modifications, functional requirements, and differential processing or target gene availability. Their dysregulation has been closely linked to cancers and various diseases, establishing miRNAs as promising non-invasive diagnostic and prognostic biomarkers. For instance, circulating miRNAs in blood or other body fluids are increasingly being utilized for early cancer detection or disease outcome prediction [22,23,24]. Therapeutically, miRNA mimics, which restore the function of downregulated tumor-suppressor miRNAs, and antagomiRs, antisense oligonucleotides designed to inhibit oncogenic miRNAs, have shown promise in cancer treatment [25,26,27,28,29,30]. RSF1 has been known as a target of multiple microRNAs. For example, in cervical cancer, miR-503-5p enhances cisplatin resistance by downregulating RSF1 expression [31], while miR-185-5p also influences cisplatin resistance and angiogenesis by regulating RSF1 [32]. In bladder cancer, miR-154 reduces RSF1 expression, thereby inhibiting tumor growth and metastasis [33]. In non-small cell lung cancer, miR-490-3p suppresses malignant progression by decreasing RSF1 expression, affecting cell proliferation and apoptosis [34]. In colon cancer, miR-129-5p exerts an anticancer effect by targeting RSF1, significantly impacting cell proliferation and apoptosis [35]. In ovarian cancer, miR-150-5p regulates the RSF1/NF-κB signaling pathway, increasing sensitivity to paclitaxel [36]. In gastric cancer, miR-1224-5p is also involved in RSF1 regulation, influencing cancer progression [37]. However, there have been no studies on miRNA-mediated regulation of RSF1 in esophageal cancer.

In this study, we investigated the role and underlying mechanisms of RSF1 regulation in ESCC. Our findings reveal the levels of RSF1 are significantly elevated in ESCC and inversely correlated with miR-193b-3p expression. Through bioinformatic analyses and luciferase reporter assays, we identified RSF1 as a direct target of miR-193b-3p. We further demonstrated the tumor-suppressive effects of miR-193b-3p on ESCC cell proliferation, migration, and invasion. Conversely, tumorigenesis was largely restored by re-expression of RSF1. These results establish that miR-193b-3p exerts its regulatory roles in ESCC by targeting the RSF1. In conclusion, our study provides novel insights into the miRNA-193b-3p/RSF1 axis as a potential therapeutic target of this aggressive malignancy.

## 2. Materials and Methods

### 2.1. Bioinformatics Analysis

**Figure 1:**
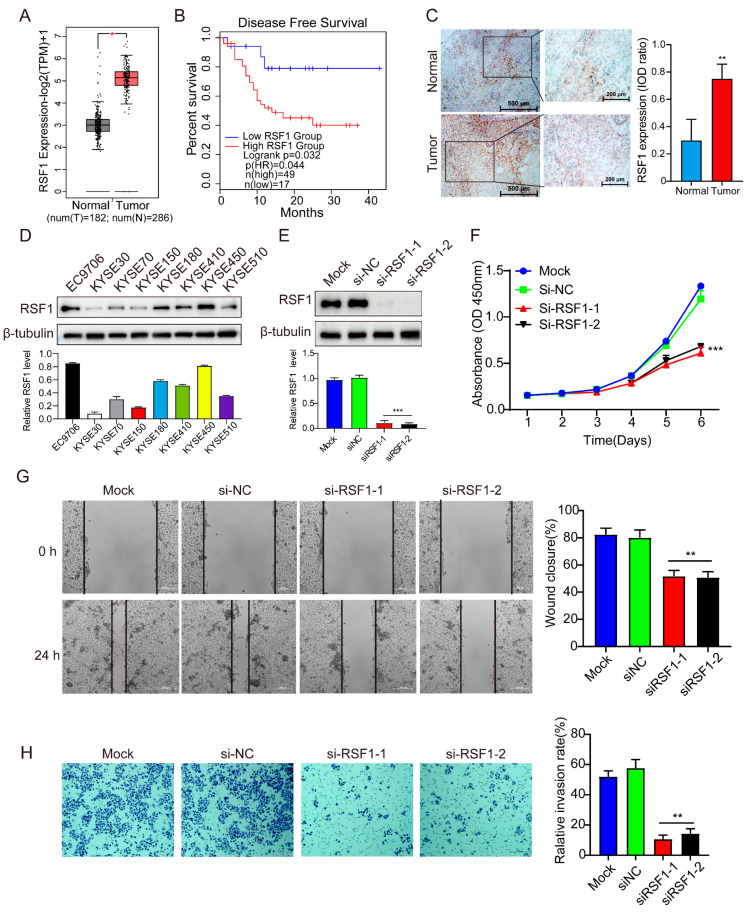
RSF1 is upregulated in ESCC and regulates key malignant phenotypes. (**A**) RSF1 mRNA levels are significantly elevated in ESCC tissues compared to adjacent non-cancerous tissues. * *p* < 0.05. (**B**) Kaplan–Meier survival analysis reveals that high RSF1 expression correlates with reduced survival in ESCC patients. (**C**) Tissue microarray immunohistochemistry showing RSF1 protein levels in ESCC and paired adjacent normal tissues. The scores (proportion and signal strength) of RSF1-positive cells are quantified and shown at right. ** *p* < 0.01. (**D**) Immunoblot analysis showing RSF1 protein levels across a panel of ESCC cell lines. (**E**) Immunoblot confirming the knockdown efficiency of si-RSF1 in KYSE450 cells. *** *p* < 0.001. (**F**) CCK-8 assays evaluating the impact of RSF1 knockdown or control si-NC on KYSE450 proliferation. *** *p* < 0.001. (**G**) Wound-healing assay demonstrating suppressed migration in RSF1-knockdown or control Si-NC on KYSE-450 cells. Scale bar = 200 μM. ** *p* < 0.01. (**H**) Transwell assay showing reduced migration and invasion capabilities in RSF1-knockdown or control Si-NC on KYSE-450 cells. Scale bar = 200 μM ** *p* < 0.01.

**Figure 2:**
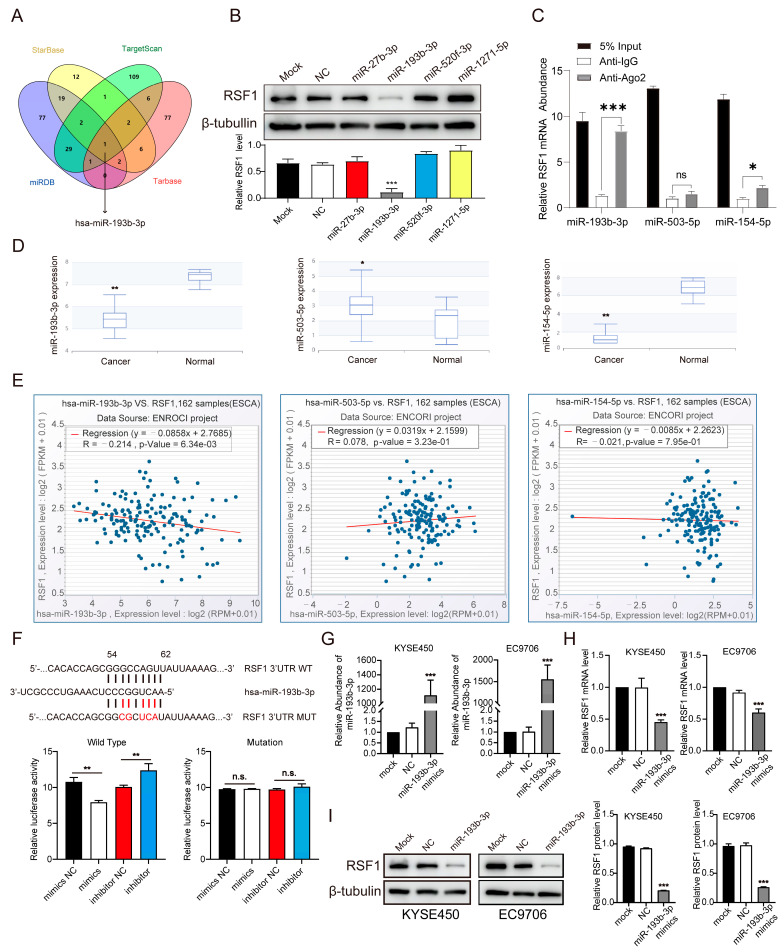
miR-193b-3p negatively correlates with RSF1 levels in ESCC. (**A**) Venn diagram illustrating the overlap of potential miRNAs targeting RSF1, as predicted from four database. (**B**) Changes in RSF1 protein levels were assessed by immunoblots after transfecting KYSE450 cells with miR-27b-3p, miR-193b-3p, miR-520f-3p, or miR-1271-5p mimics. *** *p* < 0.001. (**C**) RIP and RT-qPCR were used to assess the enrichment of RSF1 mRNA in IgG or Ago2 immunoprecipitates. IgG served as a negative control and 5% Input was used as a positive control. * *p* < 0.05, *** *p* < 0.001. (**D**) Expression levels of miR-193b-3p, miR-503-5p, and miR-154-5p in esophageal cancer tissues and adjacent normal tissues. * *p* < 0.05, ** *p* < 0.01. (**E**) Correlation analysis of miR-193b-3p, miR-503-5p, and miR-154-5p levels with RSF1 RNA levels in ESCC tissues. (**F**) Predicted binding sites of miR-193b-3p in the 3′ UTR of the wild-type RSF1 mRNA and the corresponding mutated sequences (top panels). Luciferase assay results in HEK293T cells show differential activity after co-transfection with RSF1-WT or RSF1-MUT constructs and miR-193b-3p mimics or inhibitor or negative controls (bottom panels). ** *p* < 0.01. (**G**) Relative abundance of miR-193b-3p determined by RT-qPCR analysis in KYSE450 and EC9706 cells transfected with miR-193b-3p mimics and negative controls. *** *p* < 0.001. (**H**) RSF1 mRNA levels in KYSE450 and EC9706 cells were measured by RT-qPCR analysis after transfection with miR-193b-3p mimics. *** *p* < 0.001. (**I**) RSF1 protein levels in KYSE450 and EC9706 cells were examined by immunoblot assays after transfection with miR-193b-3p mimics. Representative images are shown at left and quantifications are shown at right. *** *p* < 0.001.

The GEPIA 2 database (http://gepia2.cancer-pku.cn/ (accessed on 15 August 2023)) was used to analyze RSF1 expression levels in normal and esophageal cancer tissues (Figure 1A), as well as to evaluate the correlation between RSF1 expression levels and disease-free survival (DFS) (Figure 1B) [38]. To identify potential miRNAs that may regulate RSF1, we performed a comprehensive search across four miRNA prediction databases: TargetScan (https://www.targetscan.org/vert_80/ (accessed on 15 August 2023)) [39], Starbase (https://rnasysu.com/encori/ (accessed on 15 August 2023)) [40], Tarbase (http://carolina.imis.athena-innovation.gr/diana_tools/web/index.php?r=tarbasev8%2Findex/ (accessed on 15 August 2023)) [41], and miRDB (https://mirdb.org/ (accessed on 15 August 2023)) [42,43]. A Venn diagram was created to intersect the results from these databases, leading to the identification of several miRNAs, including miR-193b-3p, as the most likely miRNAs to regulate RSF1 (Figure 2A). Subsequently, we analyzed the expression levels of these miRNAs in esophageal cancer tissues and adjacent non-cancerous tissues using data from the dbDEMC database (dbDEMC 3.0, https://www.biosino.org/dbDEMC/index (accessed on 15 August 2023)) (Figure 2D) [44]. We further investigated the correlation between these miRNAs and RSF1 expression in esophageal cancer using the starBase database (Figure 2E). Finally, we employed the TargetScan database to predict the interaction sites between miR-193b-3p and RSF1, confirming the specific sequence through which miR-193b-3p modulates RSF1 expression (Figure 2F).

### 2.2. Immunohistochemistry Assay

After deparaffinization, rehydration, and antigen retrieval, tumor tissue slides were incubated overnight at 4 °C with a rabbit polyclonal antibody against human RSF1 (diluted 1:100; Abcam, Cambridge, UK, ab109002). The following day, RSF1 expression was detected using DAB chromogenic staining, followed by counterstaining with hematoxylin to visualize cell nuclei. The slides were then dehydrated, cleared with xylene, and mounted for long-term preservation. RSF1 expression was quantitively assessed by evaluating both the percentage of positively stained cells and the staining intensity. A combined score, integrating the IOD ratio (Integrated Optical Density), which includes both the intensity of the signals and the percentage of positive cells, was calculated to provide a comprehensive measurement of RSF1 expression levels (Figure 1C). The IOD ratio is determined by multiplying the signal intensity with the area of positive staining, providing an integrated assessment of the protein expression.

### 2.3. Cell Culture

The human esophageal cancer cell lines EC9706, KYSE30, KYSE70, KYSE150, KYSE180, KYSE410, KYSE450, and KYSE510 were obtained from the Cell Bank of the Chinese Academy of Sciences (Shanghai, China). The cells were cultured in RPMI-1640 medium (Corning, NY, USA), supplemented with 10% fetal bovine serum (FBS) (Corning, NY, USA). The 293T cell line, derived from an isolate preserved in our laboratory, was cultured in DMEM (Corning, NY, USA) supplemented with 10% FBS. All cells were maintained at 37 °C in a humidified incubator with 5% CO_2_.

### 2.4. Cell Transfection Assay

miR-193b-3p mimics and inhibitors, along with their respective negative controls, were chemically synthesized by Genepharma (Shanghai, China). si-RSF1 were chemically synthesized by Tsingke (Peking, China). The sequences of these oligonucleotides were as follows (Table 1):

These miRNAs or siRNAs were transfected into ESCC cells using RNATransMate (Sangon Biotech, Shanghai, China) following the manufacturer’s instruction. Specifically, 2 mL of Opti-MEM medium containing a final concentration of 25 nM miRNA mimics, inhibitors, siRNA, or their respective negative controls was mixed with 6 μL of RNATransMate and added to a 6-well plate seed with 3 × 10^5^ ESCC cells. For RSF1 and control pLVX vector transfections, 2.5 μg of plasmids were mixed with 3.5 μL of Lipofectamine 3000, 7 μL of P3000, and 2 mL of Opti-MEM before adding to a 6-well plate seeding with ESCC cells. Following transfection, the cells were incubated at 37 °C with 5% CO_2_ for 6 h, after which the media were replaced with fresh, complete media. The cells were then incubated for an additional 48 h before further analyses.

### 2.5. Immunoblots

Total proteins were extracted from cells using Radio Immunoprecipitation Assay (RIPA) lysis buffer (PC101, Epizyme, Shanghai, China), supplemented with 1 mM phenylmethanesulfonyl fluoride (PMSF; AR1179, Boster, Wuhan, China), 1 mM dithiothreitol (DTT), and 1 mM protease inhibitor cocktail (B15001.A/B15001.B, Bimake, Shanghai, China). Cells were lysed on ice for 30 min. The protein extracts were separated by 7.5% sodium dodecyl sulfate-polyacrylamide gel electrophoresis (SDS-PAGE) and transferred onto a PVDF membrane (Millipore, Billerica, MA, USA). The membrane was blocked with 5% milk at room temperature for 2 h, incubated with primary antibodies at 4 °C overnight, and then with horseradish peroxidase (HRP)-conjugated secondary antibody at room temperature for 2 h. Protein signals were visualized using chemiluminescence with an ECL kit (AR1170, Boster, Wuhan, China). Images were captured using the Amersham ImageQuant 800 system (Cytiva, Marlborough, MA, USA). The intensity of the protein bands was quantified and analyzed using ImageJ software (version 1.53t). The primary antibodies used included anti-RSF1 (1:1000, ab109002; Abcam, Cambridge, UK) and anti-β-tubulin (1:1000, ABL1030; Abbkine, Beijing, China), with β-tubulin serving as the loading control. Western blot band intensities were quantified and normalized to β-tubulin. Data are expressed as mean ± standard deviation (SD). Statistical analysis was performed using an unpaired two-tailed *t*-test for comparisons between two groups or one-way ANOVA with Tukey’s post hoc test for multiple comparisons (*n* = 3, * *p* < 0.05, ** *p* < 0.01, *** *p* < 0.001).

### 2.6. Quantitative Real-Time PCR Assay

Total microRNA was extracted using the SanPrep Column microRNA Extraction Kit (B518621, Sangon Biotech, Shanghai, China), which is specifically designed for the efficient purification of small RNAs, including mature microRNAs. Reverse transcription was performed using the miRNA 1st Strand cDNA Synthesis Kit (by stem-loop) (MR101-01, Vazyme, Nanjing, China), which employs stem-loop primers to ensure high specificity and efficiency for mature miRNA reverse transcription. Each 20 µL reaction mixture contained 500 ng of total RNA, stem-loop RT primers specific to the target miRNA and the internal control U6, along with dNTPs, reverse transcriptase, and RNase inhibitor. The reverse transcription was carried out at 42 °C for 60 min, followed by 95 °C for 5 min to inactivate the enzyme. Quantitative PCR was then performed using the miRNA Unimodal SYBR qPCR Master Mix (MQ102-01, Vazyme, Nanjing, China) on a real-time PCR system under the following cycling conditions: 95 °C for 30 s, followed by 40 cycles of 95 °C for 10 s and 60 °C for 30 s. All reactions were conducted in triplicate, and relative miRNA expression levels were calculated using the 2^−ΔΔCt^ method, normalized to U6 expression. The primers used for qRT-PCR were as follows (Table 2):

### 2.7. MicroRNA Immunoprecipitation (RIP) Assay

MiRNA/mRNA immunoprecipitation (IP) was performed using the RNA Immunoprecipitation (RIP) Kit (absin, Shanghai abs50071, CN) according to the manufacturer’s protocol. Briefly, KYSE450 cells (2 × 10^7^) were transfected with miRNA mimics (60 nM) using RNA Transmate (Sangon, E607402, CN) and incubated for 48 h. The transfected cells were then lysed in 405 μL of complete lysis buffer supplemented with a protease inhibitor cocktail and RNase inhibitors, followed by incubation on ice for 30 min. The lysates were centrifuged, and the supernatants were collected for further analysis. The supernatants were incubated overnight with RIP buffer containing either anti-Ago2 antibody (CST, Danvers, MA 2897T, US) or beads conjugated to anti-IgG as a negative control. After purifying the immunoprecipitated RNA from the magnetic beads, qRT-PCR analysis was performed to assess the relative enrichment of RSF1 mRNA.

### 2.8. Dual-Luciferase Reporter Assay

A Wild-type RSF1 3′-UTR fragment and a fragment containing mutations in the putative binding site for miR-193b-3p were synthesized and inserted into the pmiRGlo plasmid (Tsingke Biotechnology, Beijing, China) for luciferase reporter assays. The wild-type fragment included the conserved miR-193b-3p binding site, whereas the mutant fragment had this site disrupted to abolish binding. These reporter constructs were transfected into HEK293T cells alongside miR-193b-3p mimics (WT or scrambled negative control, NC) or an inhibitor (WT or scrambled negative control, NC) to evaluate the functional impact of miR-193b-3p on the RSF1 3′-UTR. Luciferase activities were measured using a luciferase assay kit (RG029S/M, Beyotime Biotechnology, Shanghai, China), providing a quantitative readout of the regulatory effects of miR-193b-3p on RSF1 3′-UTR.

### 2.9. Cell-Counting Assay

Cells were seeded into 96-well plates at a density of 2 × 10^3^ cells per well and allowed to adhere for 24 h under standard culture conditions. Following the adhesion period, 10 µL of Cell Counting Kit-8 (CCK-8) reagent (C0037, Beyotime Biotechnology, Shanghai, China) was added to each well in a light-protected environment. The plates were then incubated at 37 °C for 6 h, allowing for the reduction in the CCK-8 reagent to a formazan product, which is directly proportional to the number of viable cells. After incubation, the culture medium was replaced with a fresh medium. At each designated time point (6 h post-transfection and at subsequent 24 h intervals), 10 µL of CCK-8 reagent was again added to each well. The plates were incubated at 37 °C for an additional 2 h to facilitate the generation of formazan. The optical density (OD) at 450 nm was then measured using a Tecan Infinite F200 microplate reader (Crailsheim, Kronach Germany). The OD values, reflecting the amount of formazan produced, were used to quantify cell viability and proliferation at each time point.

### 2.10. Colony Formation Assay

Cells were seeded into 6-well plates at a density of 800 cells per well and incubated for 14 days to allow colony formation. After incubation, the cells were washed three times with phosphate-buffered saline (PBS) to remove the residual culture medium. To fix the colonies, 4% paraformaldehyde was added, and the cells were incubated at room temperature for 30 min. Following fixation, the cells were stained with 0.1% crystal violet solution for 30 min to enable clear visualization of the colonies. Excess dye was removed by gently rinsing the wells with PBS. Colonies containing more than 50 cells were counted under a microscope, and the total number of colonies was recorded.

### 2.11. EdU Incorporation Assay

To assess cell proliferation, an EdU incorporation assay was used to detect newly synthesized DNA in proliferating cells. Briefly, 48 h post-transfection, cells were incubated with 0.5 mL of culture medium containing 10 μM EdU (5-ethynyl-2′-deoxyuridine) for 2 h to allow incorporation into DNA during the S phase of the cell cycle. After EdU labeling, cells were fixed with 4% paraformaldehyde at room temperature for 30 min, followed by 3 to 5 washes with PBS to remove residual fixative. Cell membranes were then permeabilized using 0.5% Triton X-100 for 10 min at room temperature, followed by additional PBS washes. Detection of incorporated EdU was achieved via a copper(I)-catalyzed azide–alkyne cycloaddition (“click” chemistry) reaction. In this reaction, a fluorescent azide-modified oligonucleotide probe (Alexa Fluor 594-azide) binds covalently and specifically to the terminal alkyne group of EdU, forming a stable triazole linkage. This enables precise visualization of newly synthesized DNA without the need for DNA denaturation, offering superior sensitivity and preserving cellular structure. After the labeling reaction, cells were counterstained with DAPI and observed under a fluorescence microscope (Shinjuku, Tokyo, Japan). Images were captured for subsequent quantification and statistical analysis.

### 2.12. Wound-Healing Assay

Cells were seeded into six-well plates and cultured until reaching approximately 90% confluence. A scratch was created across the center of each well using a 10 μL pipette tip, forming a wound in the cell monolayer. Afterward, the cells were washed twice with PBS to remove any detached cells and debris. Cells were then cultured in a serum-free medium to inhibit further proliferation, focusing on cell migration. Over a 24 h incubation period, cells at the edges of the wound migrated to close the gap. At the end of the incubation, the wound area was observed under a microscope, and images of the scratched region were captured to document the extent of cell migration and wound closure. The width of the scratch was measured at multiple points, and the migration rate was quantitatively assessed by comparing the initial and final scratch widths.

### 2.13. Transwell Assay

The migration and invasion capacities of ESCC cells were evaluated using Transwell chambers with an 8 μm pore size membrane (353097, BD Falcon, Franklin Lakes, NJ, USA). For the migration assay, cells were seeded into the upper chamber without Matrigel. For the invasion assay, the upper chamber was pre-coated with Matrigel to simulate the extracellular matrix. The lower chamber was filled with a medium containing 30% fetal bovine serum (FBS) to serve as a chemoattractant. Following transfection, ESCC cells were cultured for 24 h before being seeded in the upper chamber with a serum-free medium. The chambers were incubated at 37 °C in a 5% CO_2_ atmosphere to allow cell migration or invasion through the membrane. After an additional 48 h incubation period, cells that had migrated or invaded the lower surface of the membrane were fixed with methanol and stained with 0.1% crystal violet for visualization. The stained cells on the lower surface of the membrane were observed under a microscope, and images were captured to document the extent of migration and invasion. The number of migrated or invasive cells was quantified by counting the stained cells in the lower chamber using Image Pro Plus (version 6.0) software.

### 2.14. Cell Apoptosis Analysis

For flow cytometric analysis, cells were cultured and treated under defined conditions before harvesting and preparation for staining. For apoptosis detection, cells were stained with Annexin V-FITC and PI using the Annexin V-FITC Apoptosis Detection Kit (San Jose, CA, USA). This staining enabled the differentiation of viable, early apoptotic, and late apoptotic/necrotic cells. After staining, cells were analyzed by flow cytometry, with appropriate gating applied to exclude debris and dead cells. Flow cytometric data were processed using FlowJo (version 10.8.1) software to quantify apoptotic stages.

### 2.15. Xenograft Assay

BALB/C BALB/C nude mice (20 females, aged 5 weeks, weighing 16–20 g) were obtained from SPF Animals (Beijing, China) and acclimatized for one week prior to the experiment. To establish the xenograft tumor model, 200 μL of sterile medium containing 2 × 10^6^ KYSE-450 cells was subcutaneously injected into the right axillary region of each mouse.

After tumor implantation, mice were randomly divided into two equal groups (*n* = 10 per group). The experimental group received injections of AgomiR-193b-3p (GenePharma, Shanghai, China), a chemically modified, cholesterol-conjugated, double-stranded RNA oligonucleotide designed to mimic endogenous miR-193b-3p and enhance its expression in vivo. The control group received AgomiR-NC, a nonspecific scrambled sequence with the same chemical modifications, which was used to control for sequence-independent effects. Both AgomiRs were delivered via direct intratumoral injection every three days for three weeks. These chemically modified AgomiRs are not naked oligonucleotides; they are designed for enhanced stability, cellular uptake, and in vivo delivery without requiring additional transfection reagents or carriers.

Tumor size was measured every three days using a Vernier caliper, and tumor volume was calculated using the formula volume = ½ × (length × width^2^). At the end of the treatment period, mice were humanely euthanized, and tumors were surgically harvested for histological and molecular analyses. All animal experiments were conducted in accordance with the ethical guidelines of Henan University and approved by the Institutional Animal Care and Use Committee (IACUC Number: HUSOM2025-007).

### 2.16. Statistical Analysis

All experiments were independently conducted in triplicate three times to ensure reliability and reproducibility, with strict control of variables to minimize systematic errors. Statistical analyses were performed using the Student’s *t*-test for pair-wise comparisons and one-way analysis of variance (ANOVA) for multi-group comparisons. A *p* value < 0.05 was considered statistically significant, while *p* > 0.05 indicated no significant difference between groups. All statistical analyses were conducted using specialized software to ensure precise calculations and reliable results.

## 3. Results

### 3.1. RSF1 Levels Are Elevated in ESCC and Correlate with Disease Progression

We first explored whether RSF1 plays a role in ESCC. Utilizing the GEPIA 2 database (http://gepia2.cancer-pku.cn/ (accessed on 15 August 2023)), we examined if there is an association between RSF1 expression and disease-free survival (DFS) in ESCC patients. The analyses revealed a significant upregulation of RSF1 mRNA expression in ESCC cancer tissues compared to adjacent normal tissues (Figure 1A). Kaplan–Meier survival analysis further demonstrated that elevated RSF1 expression negatively correlates with DFS in ESCC patients (Figure 1B). To validate these findings, we employed tissue microarray immunohistochemistry to compare RSF1 protein levels in ESCC tumor tissues with adjacent normal tissues. The results confirmed that RSF1 protein levels were significantly higher in tumor tissues relative to adjacent normal counterparts (Figure 1C). Statistical analyses correlating RSF1 expression levels with patient clinicopathological features are presented in Supplementary Table S1. Collectively, these findings indicate that elevated RSF1 levels in ESCC are associated with disease progression and poor patient survival outcomes.

To investigate further the role of RSF1 in ESCC, we measured RSF1 protein levels across a panel of ESCC cell lines, including EC9706, KYSE30, KYSE70, KYSE150, KYSE180, KYSE410, KYSE450, and KYSE510, using immunoblots. RSF1 protein levels were markedly elevated in EC9706 and KYSE450 cells compared to other cell lines (Figure 1D). To assess the functional significance of elevated RSF1 expression in ESCC, we silenced RSF1 with siRNA in KYSE450 cell (Figure 1E) and EC9706 cell (Supplementary Materials, Figure S1A), and analyzed ESCC cell proliferation with CCK-8 assays. The results revealed that RSF1 knockdown significantly impaired cell proliferation compared to control groups (mock and si-NC) (Figure 1F and Supplementary Materials, Figure S1B). Wound-healing assays and Transwell assays demonstrated that RSF1 knockdown significantly inhibited the migration and invasion capabilities of KYSE450 ESCC cells (Figure 1G,H and Supplementary Materials, Figure S1C,D). These findings underscore the critical role of RSF1 in regulating ESCC cell proliferation, migration, and invasion.

### 3.2. Identification of miR-193b-3p as a Critical Regulator of RSF1 in ESCC

To identify potential miRNAs that regulate RSF1 in ESCC, we searched four miRNA prediction databases (Target Scan, Starbase, Tarbase, and miRDB) and identified miR-193b-3p, which was predicated by all four databases, as well as miR-27b-3p, miR-520f-3p, and miR-1271-5p, that were predicted from three databases (Figure 2A). To determine whether these miRNAs target RSF1, we transfected KYSE450 cells with miRNA mimics and examined the effect on RSF1 protein levels. Compared to untreated KYSE450 cells (Mock) and the negative control group (NC) transfected with scrambled miRNA sequences, only the miR-193b-3p mimic significantly reduced RSF1 protein levels, while the other miRNAs had minimal effects (Figure 2B). To determine whether miR-193b-3p directly binds to RSF1 mRNA, we performed a microRNA RNA immunoprecipitation (miRNA-RIP) assay. The Ago2-miRNA co-immunoprecipitation (co-IP) assay enriches miRNA-mRNA complexes bound to Argonaute2 (Ago2), the catalytic core of the RNA-induced silencing complex (RISC). This technique enables the identification of functional miRNAs that specifically bind their target mRNA sequences, as Ago2 selectively incorporates miRNAs exhibiting perfect or near-perfect complementarity to cognate targets. KYSE450 cells were transfected with miR-193b-3p mimics, miR-503-5p, or miR-154-5p mimics; the latter two miRNAs were previously reported to regulate RSF1 [31,33]. RSF1 mRNA enrichment in Ago2-containing granules was then examined. Compared to IgG controls, miR-193b-3p mimics significantly enriched RSF1 mRNA in Ago2 granules (8-fold enrichment, *p* < 0.001). In contrast, the miR-154-5p mimics resulted in only a modest increase (1-fold, *p*= 0.042), while miR-503-5p mimics showed no significant enrichment (*p* = 0.714). These results indicate that miR-193b-3p exhibits the strongest binding affinity to RSF1 mRNA in ESCC cells (Figure 2C). Further analysis of ESCC tissues revealed that miR-193b-3p and miR-154-5p expression levels were significantly downregulated compared to adjacent normal tissues, whereas miR-503-5p expression was markedly upregulated (Figure 2D). However, only miR-193b-3p and RSF1 levels demonstrated a significant inverse relationship in Correlation analyses in ESCC, whereas no significant correlation was observed between RSF1 and miR-503-5p or miR-154-5p (Figure 2E). To assess further the apparent predominance of miR-193b-3p in regulating RSF1 in esophageal cancer, we evaluated the expression levels and correlations of other RSF1-regulating miRNAs previously reported in other tumors. Our analyses revealed that miR-1224-5p [37], miR-150-5p [36], and miR-185-5p [32] were significantly upregulated in esophageal cancer tissues, contrary to a suspected inhibitory role in regulating RSF1 expression. Indeed, none of these miRNAs showed a significant correlation with RSF1 expression (Supplementary Materials, Figure S2A–C). Although miR-129-5p [35] was markedly downregulated in esophageal cancer, it exhibited a weak positive correlation with RSF1 (Supplementary Materials, Figure S2D). Based on these observations, we propose that miR-193b-3p directly and negatively regulates RSF1 levels in ESCC.

To validate this hypothesis, we performed dual luciferase reporter assays. The putative wild-type (WT) and mutated (MUT) binding sites of miR-193b-3p within the RSF1 3′-untranslated region (UTR) were cloned into reporter constructs. Co-transfection of HEK293T cells with RSF1-3′UTR-WT or RSF1-3′UTR-MUT constructs and miR-193b-3p mimics demonstrated a significant reduction in luciferase activity in cells transfected with the WT construct, but not with the MUT construct. In contrast, upon co-transfection of miR-193b-3p inhibitor, the luciferase activity increased, but only when the WT construct was used, not the mutated construct. Scrambled oligonucleotides of the mimics or inhibitor oligonucleotides were used as NC control groups to serve as baselines for validating the specificities of the target sequences (Figure 2F). These findings strongly suggest that miR-193b-3p directly binds to the RSF1 3′UTR. Next, we transfected miR-193b-3p mimics in two ESCC cell lines, EC9706 and KYSE450, that had low levels of endogenous miR-193b-3p (Figure 2G). Quantitative RT-PCR (qRT-PCR) and immunoblot blot analyses showed that miR-193b-3p mimics significantly reduced both the mRNA and protein levels of RSF1 in these cells (Figure 2H,I). These results conclusively demonstrate that RSF1 is a direct target of miR-193b-3p and that miR-193b-3p negatively regulates RSF1 levels in ESCC.

### 3.3. Suppression of miR-193b-3p Increases RSF1 Protein Levels and Enhances ESCC Progression

**Figure 3:**
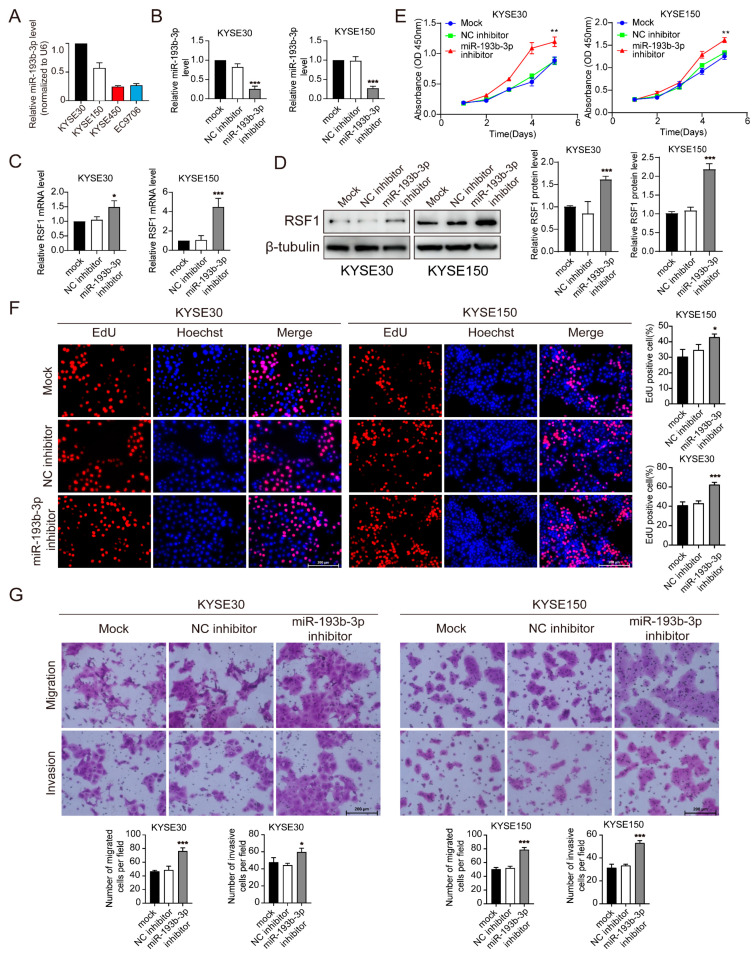
Suppression of miR-193b-3p expression promotes the progression of ESCC. (**A**) RT-qPCR analyses of miR-193b-3p expression levels in ESCC cell line KYSE30, KYSE150, KYSE450, and EC9706. (**B**) RT-qPCR analyses showing decreased miR-193b-3p levels in KYSE30 and KYSE150 cells following transfection of miR-193b-3p inhibitor as compared to negative control (NC). *** *p* < 0.001. (**C**) RT-qPCR analyses demonstrating increased RSF1 mRNA levels in KYSE30 and KYSE150 cells following transfection with miR-193b-3p inhibitors. * *p* < 0.05, *** *p* < 0.001. (**D**) Immunoblot assays showing elevated RSF1 protein levels in KYSE30 and KYSE150 cells after transfection of miR-193b-3p inhibitor. Representative images are shown at left and quantifications are shown at right. *** *p* < 0.001. (**E**) CCK-8 assays showing enhanced proliferation of KYSE30 and KYSE150 cells treated with miR-193b-3p inhibitor as compared to NC. ** *p* < 0.01. (**F**) EdU assays revealing increased proliferation ability of KYSE30 and KYSE150 cells after transfection of miR-193b-3p inhibitor compared to NC. Representative images are shown at left and quantifications are shown at right. * *p* < 0.05, *** *p* < 0.001. (**G**) Transwell assays revealing significantly increased migration and invasion in KYSE30 and KYSE150 cells following transfection of miR-193b-3p inhibitor. Representative images are shown at top and quantifications are shown at bottom. * *p* < 0.05, *** *p* < 0.001.

To assess further the relationship between miR-193b-3p and RSF1 protein levels, RT-qPCR assays were performed to determine the relative abundance of miR-193b-3p in four ESCC cell lines exhibiting different RSF1 levels. We selected KYSE30 and KYSE150, which have low RSF1 levels, and KYSE450 and EC9706, which have high RSF1 levels (see Figure 1D). The results revealed that miR-193b-3p levels were markedly higher in KYSE30 and KYSE150 cells compared to KYSE450 and EC9706 cells, consistent with an inverse correlation between miR-193b-3p and RSF1 in ESCC cells (Figure 3A). To substantiate the interpretation, we transfected KYSE30 and KYSE150 cells with an miR-193b-3p inhibitor to suppress miR-193b-3p production. The inhibitor significantly reduced miR-193b-3p levels in both cell lines (Figure 3B) while markedly increasing levels of both RSF1 mRNA and protein (Figure 3C,D), supporting our conclusion.

To evaluate the functional impact of miR-193b-3p suppression on ESCC phenotypes, CCK-8 and EdU assays were performed. Results showed a significant increase in the proliferation of KYSE30 and KYSE150 cells transfected with the miR-193b-3p inhibitor compared to the mock (Mock) and negative control (NC inhibitor) groups (Figure 3E). EdU incorporation assays revealed a significant increase in fractions of proliferative cells following miR-193b-3p inhibitor transfection (Figure 3F). Transwell migration and invasion assays demonstrated that suppression of miR-193b-3p significantly enhanced the migration and invasion capabilities of KYSE30 and KYSE150 cells (Figure 3G). Together, these findings confirm the role of miR-193b-3p in negatively regulating ESCC progression.

### 3.4. Elevation of miR-193b-3p Inhibits ESCC Cell Proliferation, Migration, and Invasion

**Figure 4:**
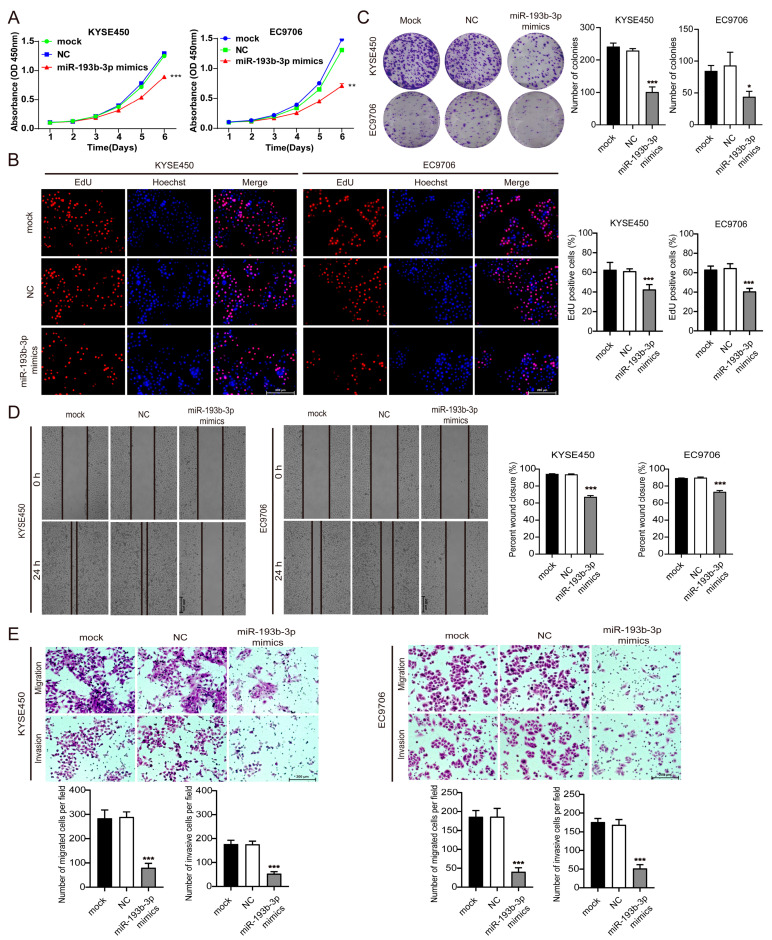
miR-193b-3p inhibits ESCC cell proliferation, invasion, and migration. (**A**) CCK-8 assays showing the decreased proliferation of KYSE450 and EC9706 cells following transfection with miR-193b-3p mimics compared to mock or negative control (NC) mimics. ** *p* <0.01, *** *p* < 0.001. (**B**) Edu assay demonstrating the reduced proliferation ability of KYSE450 and EC9706 cell lines transfected with miR-193b-3p mimics compared to NC mimics. Representative images are shown at left and quantifications are shown at right. *** *p* < 0.001. (**C**) Colony formation assay showing impaired colony-forming ability in KYSE450 and EC9706 cell lines after transfection with miR-193b-3p mimics compared to mock and NC mimics. Representative images are shown at left and quantifications are shown at right.* *p* <0.05, *** *p* < 0.001. (**D**) Wound-healing assay showing reduced migration ability of two ESCC cell lines expressing high levels of RSF1 after transfection with miR-193b-3p mimics compared to mock transfection and NC mimics. Representative images are shown at left and quantifications are shown at right. *** *p* < 0.001. (**E**) Transwell assays demonstrating significant reductions in migration and invasion capacities of the same two ESCC cell lines following transfection with miR-193b-3p mimics compared to mock transfection and NC mimics. Representative images are shown on the top and quantifications are shown in the bottom. *** *p* < 0.001.

To investigate the role of miR-193b-3p in regulating ESCC cell growth and survival, we transfected miR-193b-3p mimics or negative controls into KYSE450 and EC9706 cells, cell lines with high endogenous RSF1. Transfection of miR-193b-3p mimics significantly reduced their proliferation rates compared to mock transfection and negative controls, as assessed by CCK-8 assays (Figure 4A). EdU incorporation assays confirmed a marked decrease in the fraction of cells in a proliferative state (Figure 4B). Moreover, the colony-forming ability of KYSE450 and EC9706 cells was significantly impaired (Figure 4C). These findings suggest that miR-193b-3p suppresses ESCC cell proliferation. Furthermore, wound-healing assays revealed that the migration of KYSE450 and EC9706 cells was significantly reduced after elevating miR-193b-3p with mimics compared to the mock or negative control (Figure 4D). Transwell assays demonstrated that both migration and invasion were also decreased, but not the mock or the negative control (Figure 4E). Flow cytometry analysis also showed that elevating miR-193b-3p increased cell apoptosis significantly (Supplementary Materials, Figure S3). Taken together, these results revealed that elevated levels of miR-193b-3p suppress ESCC cell proliferation, migration, and invasion while promoting apoptosis; thus, miR-193b-3p acts as a tumor suppressor in ESCC.

### 3.5. miR-193b-3p Inhibits ESCC Tumor Growth In Vivo

**Figure 5:**
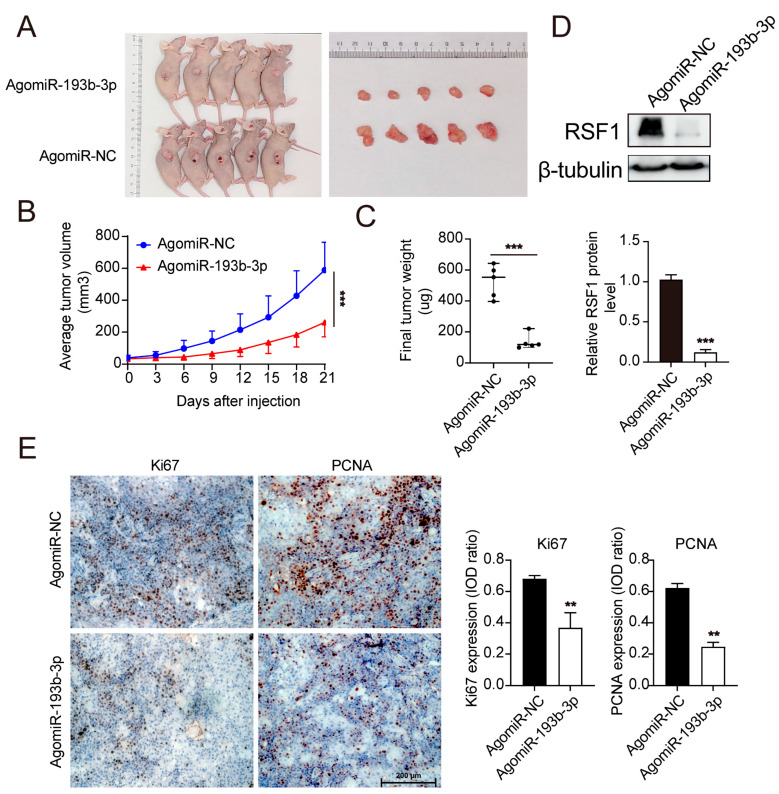
miR-193b-3p inhibits tumor growth and proliferation in vivo. (**A**) Representative images of xenograft tumors dissected from nude mice treated with AgomiR-193b-3p or AgomiR-NC. (**B**) Tumor growth curves of xenograft models demonstrate significantly reduced growth in AgomiR-193b-3p-treated group compared to AgomiR-NC controls (*n* = 5). *** *p* < 0.001. (**C**) Tumor weights from the xenograft formation assay indicate a significant reduction in tumor size in the AgomiR-193b-3p-treated group compared to AgomiR-NC controls. *** *p* < 0.001. (**D**) Immunoblot analysis of RSF1 protein levels in tumor tissues reveals a significant decrease in RSF1 expression in the AgomiR-193b-3p-treated group compared to controls (*n* = 5). (**E**) Immunohistochemical (IHC) staining of Ki-67 (left panel) and PCNA (right panel) in xenograft tumors reveals a marked reduction in cells positive for the proliferation marker expression in the AgomiR-193b-3p-treated group compared to AgomiR-NC controls. The IOD ratio is shown at right. Scale bar are indicated. *n* = 5 for each group. ** *p* < 0.01.

To evaluate the effect of miR-193-3p on ESCC tumor growth in vivo, we established a subcutaneous xenograft mouse model using KYSE450 cells. After the xenografts were established, AgomiR-193b-3p was injected subcutaneously every three days from day 3 to 15. AgomiR-193b-3p-injection significantly slowed tumor growth, reduced tumor volume, and decreased tumor weight as compared to control groups (Figure 5A–C). To assess the effect of AgomiR-193b-3p on RSF1 levels, we extracted proteins from tumor tissues of both experimental and control groups (Figure 5D). Immunoblot demonstrated a significant reduction in RSF1 protein levels in the AgomiR-193b-3p-treated group compared to controls. Immunohistochemical (IHC) staining showed a marked reduction in the expression of Ki-67 and PCNA-positive cells in tumors from the AgomiR-193b-3p-injected group compared to controls (Figure 5E). These results indicate that AgomiR-193b-3p injection effectively reduced RSF1 protein levels and inhibited tumor cell proliferation in vivo.

### 3.6. RSF1 Is a Key Target of miR-193b-3p During ESCC Progression

**Figure 6:**
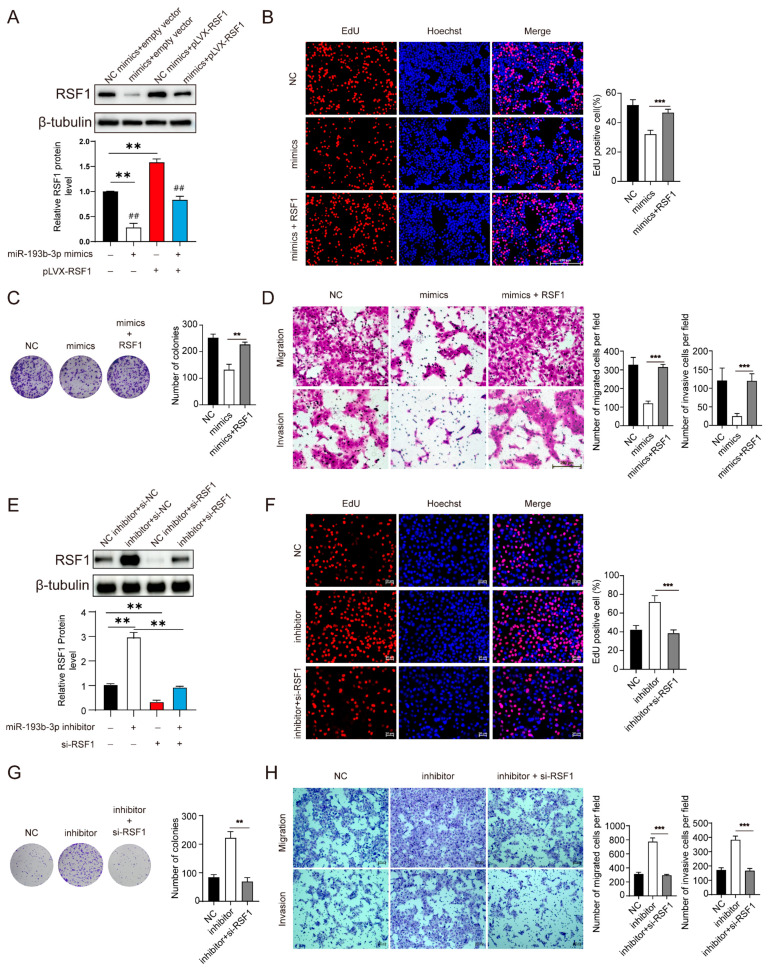
miR-193b-3p regulates ESCC progression through RSF1. (**A**) Immunoblot analyses showing the levels of RSF1 in KYSE450 transfected with miR-193b-3p mimics or an NC control along with RSF1 expression plasmid or an empty vector as indicated below. Representative images are shown at top and quantifications are shown at bottom. ** *p* < 0.01, ^##^ *p* < 0.01. (**B**) EdU assays demonstrating that exogenous RSF1 expression reversed the inhibitory effect of miR-193b-3p mimics. Representative images are shown at left and quantifications are shown at right. *** *p* < 0.001. (**C**) Colony formation assays showing that exogenous RSF1 expression restored colony-forming ability in KYSE450 cells transfected with miR-193b-3p mimics. Representative images are shown at left and quantifications are shown at right. ** *p* < 0.01. (**D**) Transwell assays indicate that exogenous RSF1 expression rescued the reduced migration and invasion of KYSE450 caused by transfected miR-193b-3p mimics. Representative images are shown at left and quantifications are shown at right. *** *p* < 0.001. (**E**) Immunoblot analyses of RSF1 levels in KYSE450 cells transfected by miR-193b-3p inhibitor or an NC control along with si-RSF1 as indicated below. Representative images are shown at the top and quantifications are shown at the bottom. ** *p* < 0.01. (**F**) EdU assay demonstrating that RSF1 silence reversed the elevated cell growth in miR-193b-3p inhibitor transfected cells reversed effects. Representative images are shown at left and quantifications are shown at right. *** *p* < 0.001. (**G**) Colony formation assays showing that silencing RSF1 in miR-193b-3p inhibitor transfected cells reversed the increased colony-forming ability. Representative images are shown at left and quantifications are shown at right. ** *p* < 0.01. (**H**) Transwell assays indicating that silencing RSF1 in miR-193b-3p inhibitor transfected cells reversed the increased migration and invasion. Representative images are shown at left and quantifications are shown at right. Scale bar = 200 μM. *** *p* < 0.001.

The above studies revealed that miR-193b-3p regulates ESCC cell proliferation, migration, invasion, and tumorigenesis and strongly suggested that RSF1 is a direct target of miR-193b-3p. To support a direct role of RSF1 in miR-193b-3p-regulated ESCC progression, we performed rescue experiments in KYSE450 cells. Immunoblotting analysis revealed that, compared to control, miR-193b-3p mimics effectively reduced RSF1 protein levels and the reduction in RSF1 protein levels was largely rescued by transfection of an RSF1 expression vector (Figure 6A). The restoration of RSF1 levels reversed the inhibitory effect of miR-193b-3p mimics on EdU incorporation (Figure 6B) and colony formation (Figure 6C). Similarly, the suppression of migration and invasion induced by miR-193b-3p mimics was also reversed by co-transfection of RSF1 (Figure 6D). Conversely, miR-193b-3p inhibition increased RSF1 levels dramatically, and the increase in RSF1 was reversed by co-transfection with siRNA against RSF1 (Figure 6E). Repression of RSF1 in miR-193b-3p inhibited cells reversed the enhanced EdU incorporation (Figure 6F) and colony formation (Figure 6G) induced by miR-193b-3p inhibitor. Furthermore, the silencing of RSF1 also effectively reversed the enhanced migration and invasion induced by the miR-193b-3p inhibitor (Figure 6H). Collectively, these findings confirmed that miR-193b-3p negatively regulates ESCC progression through RSF1, highlighting the central role of RSF1 in ESCC tumor progression.

## 4. Discussion

ESCC is a leading cause of cancer-related mortality globally, with limited therapeutic options and poor prognosis, particularly in advanced stages. In this study, we have elucidated the pivotal role of the miR-193b-3p/RSF1 axis in regulating ESCC progression. Our findings demonstrate that RSF1, a chromatin remodeling factor, is markedly upregulated in ESCC tissues relative to adjacent normal tissues and that its levels are inversely correlated to miR-193b-3p levels. Furthermore, we provide compelling evidence for functional interactions between miR-193b-3p and RSF1 in that miR-193b-3p directly targets RSF1, suppressing its oncogenic effects and inhibiting key processes such as cell proliferation, migration, invasion, and tumorigenesis. Experiments using miR-193b-3p mimics or inhibitors and, conversely, an RSF1 knockdown or expression vector in ESCC cell lines supported their opposing roles in regulating ESCC progression. These findings were further validated in vivo, where miR-193b-3p administration reduced tumor growth in a xenograft model. These studies underscore its potential as a prognostic marker and potential therapeutic target.

RSF1 primarily functions as part of the RSF (Remodeling and Spacing Factor) chromatin remodeling complex, which is critical for chromatin architecture and gene regulation [45]. Dysregulated RSF1 expression disrupts these processes, alters gene expression, and leads to oncogenesis [46,47]. In ESCC, we found that elevated RSF1 levels were strongly associated with reduced disease-free survival (DFS), suggesting its potential as a prognostic biomarker. Functional assays revealed that RSF1 silencing significantly impaired the proliferative, migratory, and invasive capacities of ESCC cells, highlighting its essential role in maintaining the malignant phenotypes of ESCC. Our findings align with studies in other cancers, such as ovarian and breast cancers [46,47], where elevated RSF1 levels drive tumor growth, resistance to therapy, and poor prognosis. Collectively, the previous and our present studies reinforce the concept that RSF1 is a universal oncogenic driver with cancer-specific nuances.

MicroRNAs are known to regulate gene expression at the post-transcriptional level, and their aberrant expression often contributes to cancer progression [48]. In this study, we identified miR-193b-3p functioning as a tumor suppressor in ESCC by directly targeting RSF1. We observed that miR-193b-3p expression was significantly lower in ESCC tissues with corresponding increased RSF1 levels compared to adjacent normal tissues. An earlier study suggested that DNA methylation might contribute to the silencing of miR-193a-3p in oral squamous cell carcinoma cell lines, suggesting an epigenetic regulation [49]. A recent research study in meningiomas has indeed demonstrated that the promoter region of miR-193b-3p is abnormally hypermethylated, leading to its transcriptional silencing [50]. These findings collectively imply that a similar epigenetic mechanism could underlie the observed downregulation of miR-193b-3p in ESCC. Future research is needed to address this issue.

Our study investigated the regulation of RSF1 by miRNAs in ESCC and identified miR-193b-3p, along with several candidates, including miR-520f-3p, miR-27b-3p, and miR-1271-5p, as potential regulators through conserved 7mer-m8 binding sites. However, only miR-193b-3p was significantly downregulated in some ESCC tissues with elevated RSF1, and transfection of its mimics into these ESCC cells significantly reduced RSF1 protein levels in KYSE450 cells. In contrast, miR-520f-3p was upregulated in high-grade ESCA, while miR-27b-3p and miR-1271-5p showed no significant expression differences in ESCC cells. Further, an miRNA-RIP analysis revealed that miR-193b-3p induced an eightfold enrichment of RSF1 mRNA in Ago2 complexes (*p* < 0.001), a significantly stronger effect than two previously reported RSF1-targeting miRNAs, miR-154-5p, and miR-503-5p. Binding site analysis showed that miR-193b-3p targets a highly conserved sequence with stronger binding affinity and inhibitory efficiency via a 7mer-m8 site, whereas miR-154-5p and miR-503-5p bind to poorly conserved sequences through 7mer-A1 sites. Although miR-154-5p is downregulated in ESCC and exhibits some tissue specificity, its targeting effect on RSF1 is weaker than that of miR-193b-3p. Taken together, these findings suggest that miR-193b-3p may serve as the primary miRNA regulator of RSF1 in ESCC.

The dual-luciferase reporter assay confirmed that miR-193b-3p binds specifically to the 3′ untranslated region (UTR) of RSF1 mRNA, thereby inhibiting its expression. This regulatory relationship between miR-193b-3p and RSF1 provides novel mechanistic insight into the molecular pathways governing ESCC progression. Furthermore, transfecting miR-193b-3p mimics into ESCC cell lines to elevate its level led to a significant reduction in RSF1 level and in cell proliferation, migration, and invasion, corroborating its role as a tumor suppressor. These findings were further validated in vivo, where miR-193b-3p mimics administration reduced RSF1 and tumor growth in a xenograft model. Taken together, our results indicate that miR-193b-3p, attributed to its strong and conserved binding affinity to RSF1 mRNA and its downregulation in ESCC, is likely the primary regulator of RSF1 expression in esophageal squamous cell carcinoma and may attenuate the aggressive behavior of ESCC, highlighting its potential as a therapeutic target [51].

The functional interaction between miR-193b-3p and RSF1 offers valuable mechanistic insights into the regulation of ESCC. RSF1 is involved in chromatin remodeling, and its dysregulation can lead to altered gene expression profiles that favor tumorigenesis. Reduction in miR-193b-3p would elevate RSF1, leading to elevated oncogene expression and inactivation of tumor suppressor pathways. This highlights the role of chromatin dynamics in cancer progression and underscores the importance of the miR-193b-3p/RSF1 axis in modulating the epigenetic landscape of ESCC. Future research would aim to address the mechanisms leading to the downregulation of miR-193b-3p miRNA.

In summary, this study highlights the critical role of the miR-193b-3p/RSF1 axis in ESCC progression. The miR-193b-3p/RSF1 axis holds great promise as both a diagnostic biomarker and a therapeutic target in ESCC. Therapeutic strategies aimed at restoring miR-193b-3p expression or inhibiting RSF1 may offer new avenues for targeted treatment, particularly for patients with advanced or metastatic disease. Given the limited efficacy of conventional therapies, targeting the miR-193b-3p/RSF1 pathway could provide a more effective and less toxic alternative.

## Figures and Tables

**Table 1:** microRNA and siRNA sequence.

Sequence Name	Sequence (5′→3′)
miR-193b-3p mimic-S	AACUGGCCCUCAAAGUCCCGCU
miR-193b-3p mimic-AS	CGGGACUUUGAGGGCCAGUUUU
miR-193b-3p mimic negative control-S	UUCUCCGAACGUGUCACGUTT
miR-193b-3p mimic negative control-AS	ACGUGACACGUUCGGAGAATT
miR-193b-3p inhibitor	AGCGGGACUUUGAGGGCCAGUU
miR-193b-3p inhibitor negative control	CAGUACUUUUGUGUAGUACAA
miR-27b-3p mimic-S	UUCACAGUGGCUAAGUUCUGC
miR-27b-3p mimic-AS	AGAACUUAGCCACUGUGAAUU
miR-520f-3p mimic-S	AAGUGCUUCCUUUUAGAGGGUU
miR-520f-3p mimic-AS	CCCUCUAAAAGGAAGCACUUUU
miR-1271-5p mimic-S	CUUGGCACCUAGCAAGCACUCA
miR-1271-5p mimic-AS	AGUGCUUGCUAGGUGCCAAGUU
miR-503-5p mimic-S	UAGCAGCGGGAACAGUUCUGCAG
miR-503-5p mimic-AS	GCAGAACUGUUCCCGCUGCUAUU
miR-154-5p mimic-S	UAGGUUAUCCGUGUUGCCUUCG
miR-154-5p mimic-AS	AAGGCAACACGGAUAACCUAUU
si-RSF1-1-S	CCAAGAAGCCCUACCGGAU
si-RSF1-1-AS	AUCCGGUAGGGCUUCUUGG
si-RSF1-2-S	GGUAGAAUGCCAGAGUACA
si-RSF1-2-AS	UGUACUCUGGCAUUCUACC

**Table 2:** Primer sequence.

Sequence Name	Sequence (5′→3′)
miR-193b-3p-F	GCGAACTGGCCCTCAAAGT
miR-193b-3p-R	AGTGCAGGGTCCGAGGTATT
U6-F	CTCGCTTCGGCAGCACA
U6-R	AACGCTTCACGAATTTGCGT
RSF1-F	GCAGATGAGGAGGAGGAGGAAGAG
RSF1-R	CTGTCACGGGCAGGCTGATTTG
GAPDH-F	TGACATCAAGAAGGTGGTGAAGCAG
GAPDH-R	GTGTCGCTGTTGAAGTCAGAGGAG

## Data Availability

The original contributions presented in this study are included in the article/Supplementary Materials. Further inquiries can be directed to the corresponding author(s).

## Supplementary Materials

The following supporting information can be downloaded at https://www.mdpi.com/article/10.3390/cells14120928/s1, Figure S1: Silencing RSF1 inhibits the malignant phenotype of EC9706 esophageal squamous cell carcinoma cells; Figure S2: Expression levels and correlations of previously reported RSF1-regulating miRNAs in esophageal cancer; Figure S3: Representative flow results and quantification of apoptosis rate in KYSE450 and EC9706 cells transfected with miR-193b-3p or NC mimics, showing a significant increase in apoptotic cells. *** *p* < 0.001. Table S1. The relationship between RSF1 expression and ESCC patients’ characteristics.
