# Supplementary material for: MicroRNA miR-193b-3p Regulates Esophageal Cancer Progression Through Targeting RSF1
Source: Cells. 2025 Jun 19;14(12):928. doi: 10.3390/cells14120928 (PMC12191309; doi:10.3390/cells14120928)
Supplement: Supplementary file 1 [file cells-14-00928-s001.zip › cells-3663980-supplementary.pdf]

Supplementary Materials

MicroRNA miR-193b-3p Regulates Esophageal Cancer Progression Through Targeting RSF1

Yao Lin[1,#], Xudong Zhao[1,2,#], Zhenhua Du[1], Zhili Jia[1], Siyu Zhou[1], Gengsheng Cao[1, *], and Hengbin Wang[3,4, *]

[1]School of Life Sciences, Henan University, Kaifeng, Henan 475004, China; [2] Department of Obstetrics and Gynecology, Li Ka Shing Faculty of Medicine, The University of Hong Kong, Hong Kong 999077, China; [3] Department of Biochemistry and Molecular Genetics, University of Alabama at Birmingham, Birmingham, AL 35294, USA; [4]Department of Internal Medicine, Division of Hematology, Oncology and Palliative Care, Massey Comprehensive Cancer Center, Virginia Commonwealth University, Richmond, Virginia 23298, USA.

# These authors contributed equally to this work.

Correspondence

*To whom correspondence should be addressed to: gscao@henu.edu.cn; hengbin.wang@vcuhealth.org.

Supplementary Figure legends

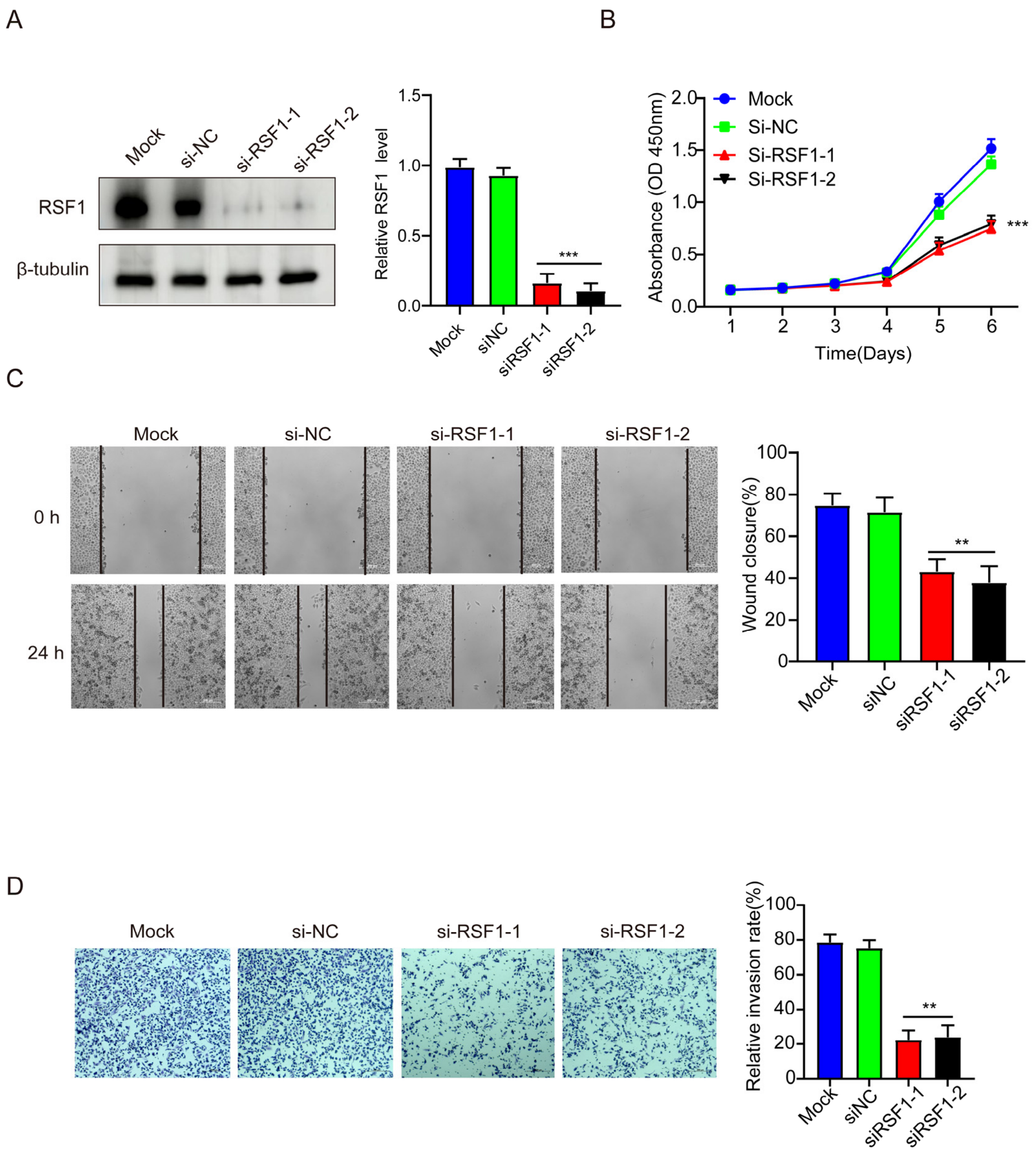

Figure S1. Silencing RSF1 inhibits the malignant phenotype of EC9706 esophageal squamous cell carcinoma cells.

- **A.** Immunoblot confirming the knockdown efficiency of si-RSF1 in EC9706 cells. ***$p < 0.001$.
- **B.** CCK-8 assays evaluating the impact of RSF1 knockdown or control si-NC on EC9706 proliferation. ***$p < 0.001$.

C. Wound healing assay demonstrating suppressed migration in RSF1-knockdown or control Si-NC on EC7906 cells. **p < 0.01.

D. Transwell assay showing reduced migration and invasion capabilities in RSF1-knockdown or control Si-NC on EC9706 cells. ***p < 0.001.

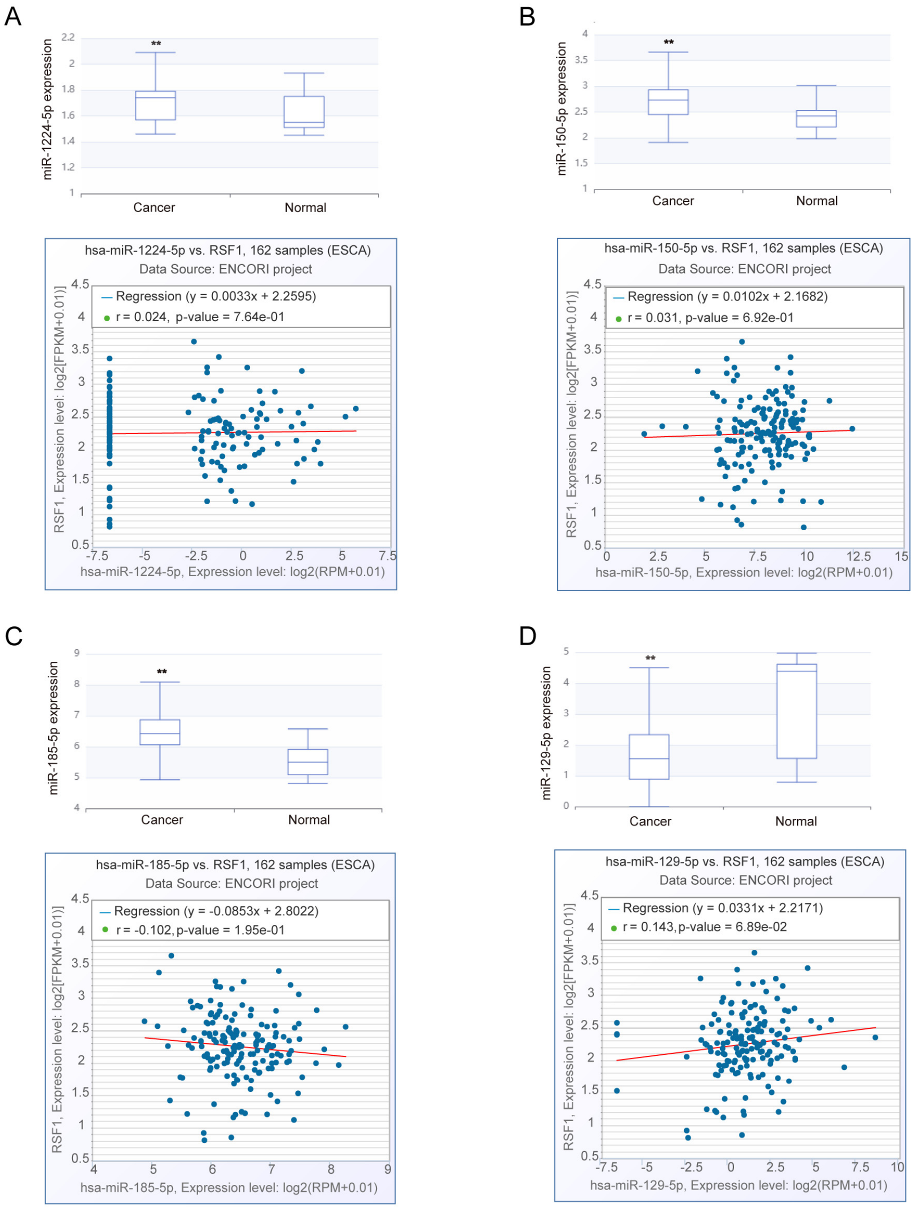

Figure S2. Expression levels and correlations of previously reported

RSF1-regulating miRNAs in esophageal cancer.

A. miR-1224-5p was significantly upregulated in esophageal cancer tissues (p value=1.86e-3) but showed no significant correlation with RSF1 expression (r=0.024, p-value=7.64e-01).

B. miR-150-5p was significantly upregulated in esophageal cancer tissues (p value=2.02e-3) but showed no significant correlation with RSF1 expression (r=0.031, p-value=6.92e-01).

C. miR-185-5p was significantly upregulated in esophageal cancer tissues (p value=7.53e-3) but showed no significant correlation with RSF1 expression (r=-0.102, p-value=1.95e-01).

D. miR-129-5p was markedly downregulated in esophageal cancer tissues (p value=1.45e-7) and exhibited a weak positive correlation with RSF1 expression (r=0.143, p-value=6.89e-02).

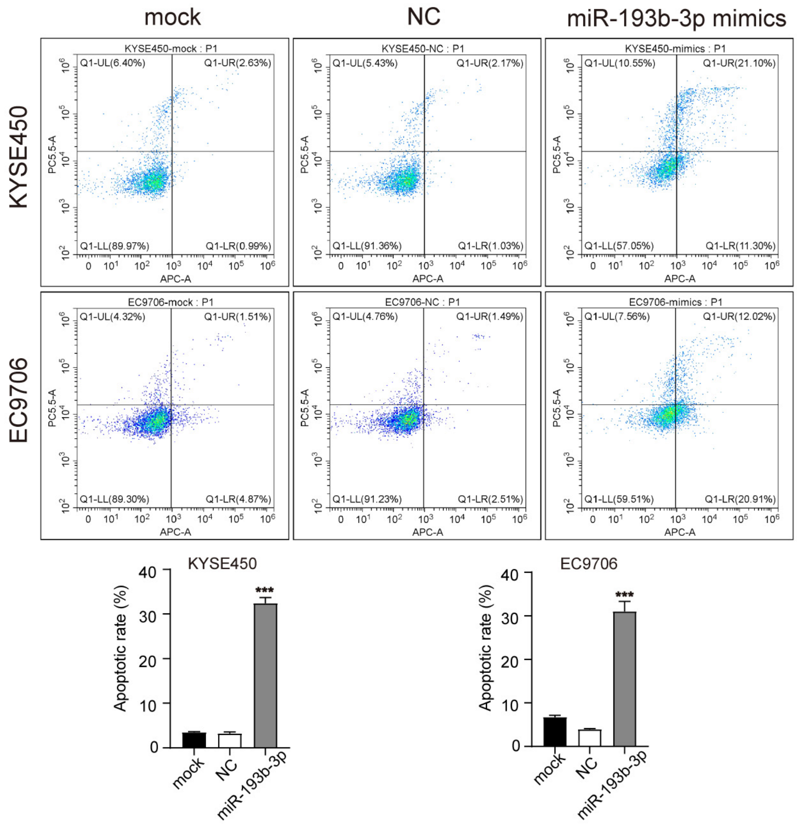

Figure S3. Representative flow results and quantification of apoptosis rate in KYSE450 and EC9706 cells transfected with miR-193b-3p or NC mimics, showing a significant increase in apoptotic cells. ***p < 0.001.

Clinico features	group	Total=15	RSF1 expression	
			low	high
Age(years)	<60	9	3（20%）	6（40%）
	≥60	6	2（13.3%）	4（26.6%）
Tumor size(cm)	<5	8	3（20%）	5（33.3%）
	≥5	7	2（13.3%）	5（33.3%）
Tumor location	Lower thoracic	6	3（20%）	3（20%）
	Middle thoracic	6	2（13.3%）	4（26.6%）
	Upper thoracic	3	1（6.6%）	2（13.3%）
Clinical stage	I	3	1（6.6%）	2（13.3%）
	I/ II	3	1（6.6%）	2（13.3%）
	II	8	2（13.3%）	6（40%）
	III	1	0（0%）	1（6.6%）

Supplementary Table S1. The relationship between RSF1 expression and ESCC patients' characteristics
